# Digital conservation: An introduction

**DOI:** 10.1007/s13280-015-0701-5

**Published:** 2015-10-27

**Authors:** René van der Wal, Koen Arts

**Affiliations:** Aberdeen Centre for Environmental Sustainability (ACES), School of Biological Sciences, Auris, University of Aberdeen, 23 St. Machar Drive, Aberdeen, AB24 3UU UK; Forest and Nature Conservation Policy Group, Wageningen University, Droevendaalsesteeg 3, 6700 AA Wageningen, the Netherlands; Centro de Pesquisa do Pantanal, Universidade Federal de Mato Grosso, Cuiabá, CEP: 78.068-360 Brazil

In the last two decades, the World Wide Web and subsequent associated developments (e.g., widely available computers, broadband, Web 2.0, the Internet of Things) have shaped old and created new modes of business, management, communication and governance. The implications for modern societies are deemed so important that some sociologists dub the current era the Digital Age (e.g., Orton-Johnson and Prior 2013). While the attributes and the dynamics of the Digital Age are subject to study in several domains, they have received relatively little attention from scholars focussing on environmental management in general and nature conservation in particular. Here, we introduce a body of work representing a new concept, ‘digital conservation,’ to start the quest for better understanding the impacts of digital innovation on nature conservation.

Nature conservation is mission driven and therefore susceptible to change over time (Haila 2012; Mace 2014). Yet, an inspection of contemporary key objectives in six international policy and practice frameworks^1^ and the mission statements of six of the largest, internationally operating non-governmental organisations and networks in nature conservation^2^ suggest the following common aims^3^:

(i) protect and restore biodiversity and natural areas;
(ii) theorise, collect and analyse data, and model and disseminate scientific findings to support systematic (evidence-based) conservation;
(iii) support (local) stakeholder-based conservation and achieve fair and democratic governance and sharing of benefits; and
(iv) promote sustainable use and management of natural resources.

Digital technology could play a key role in promoting all these aims. Indeed, the application of digital technology^4^ has rapidly gained prominence in nature conservation, in both number and diversity, and now spans a wide range of areas, including (but far from restricted to) novel monitoring tools, digital public engagement, citizen science, crowd sourcing, e-learning, e-gaming, data connectivity, and decision-making support systems. These developments at the interface of digital technology and nature conservation can be captured by the suggested umbrella term ‘digital conservation.’

As far as we are aware, the first public use of the term ‘digital conservation’ dates from 2011. It featured in a one-day workshop that brought together researchers and practitioners with an interest in digital technology, particularly with regard to citizen science for nature conservation.^5^ A year later, the term was used for a programme of work at the interface of nature conservation and computing science, funded as part of dot.rural.^6^ Working on this made us realise how keen many conservationists were on digital innovation, how numerous and diverse the existing approaches were, and how fragmented the field was. These realisations inspired us to organise the first international conference on digital innovation in nature conservation, held in May 2014 in Aberdeen.^7^ This *Ambio* Special Issue on digital conservation flows from this conference and is composed in recognition that digital technology increasingly shapes human interaction with nature, and that there is an urgent need to better understand the various dimensions of this phenomenon. We define ‘digital conservation’ as the collection of developments at the interface of digital technology and nature conservation that affect nature conservation-related goals.

Digital conservation projects and initiatives are booming. This rapid growth seems linked in with optimism among conservationists about the promise that digital conservation holds: more data, larger audiences, improved surveillance and more efficient management. Still, compared to digital technology studies in many other scientific disciplines, research into the precise impacts of digital applications on nature conservation is in its infancy. With this special issue, we hope to further kick-start such much needed research and discussion (Fig. 1).

**Fig. 1:**
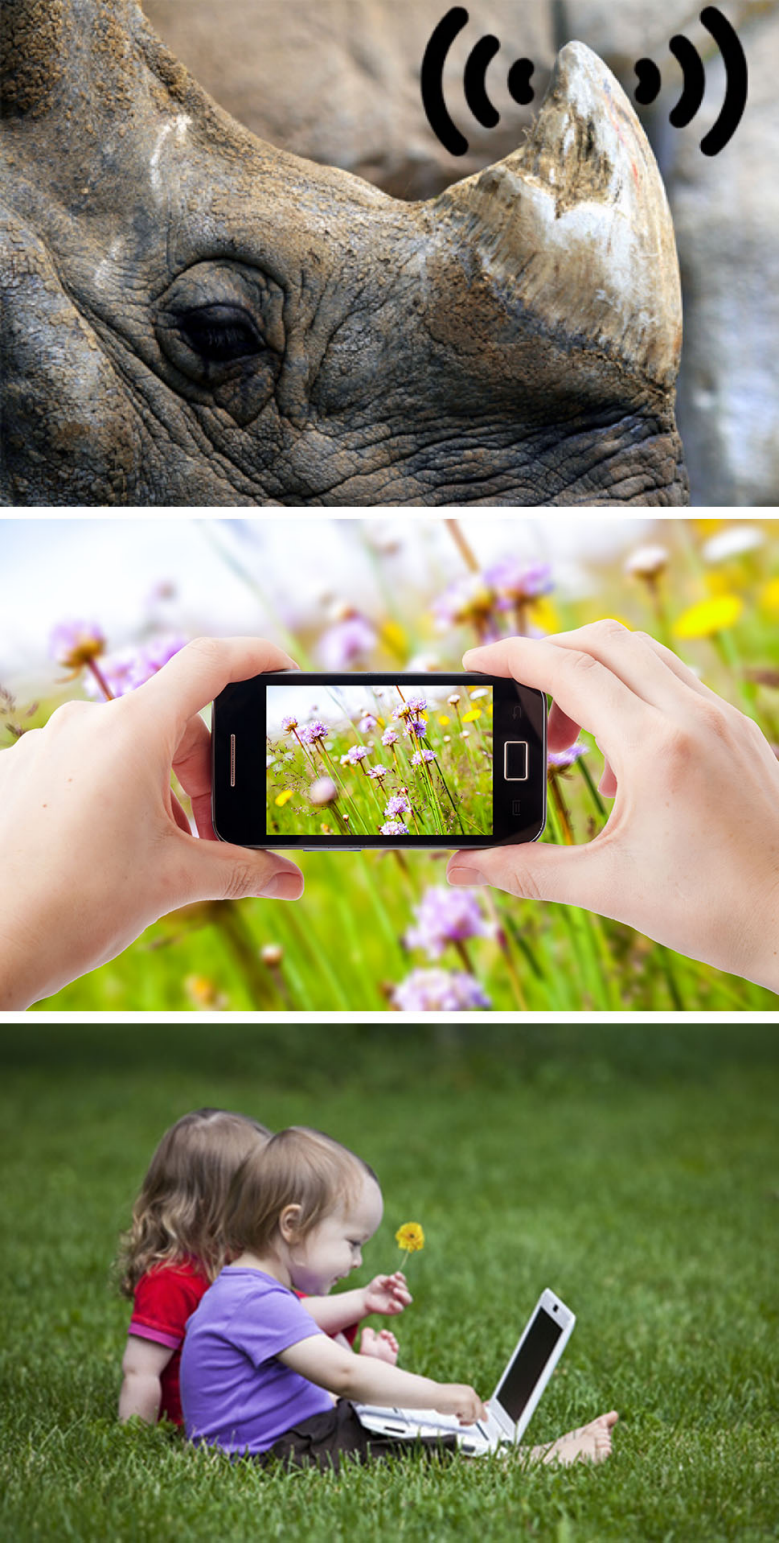
Images featuring on the flyer of the first international conference on digital innovation in nature conservation (May 2014, Aberdeen, UK) illustrating part of the landscape of this emerging field. Photo credits: *Top image* Original photo by Jim Epler/CC. The idea of the transmission symbol around the horn should be credited to Stephen Messenger (http://www.treehugger.com/clean-technology/gps-devices-installed-in-african-rhinos-horns.html); *middle* and *bottom images* obtained via Fotolia

Many types of disciplinary knowledge and skill-sets are involved in digital conservation initiatives. The very study of the phenomenon requires a similar mix of multi- and interdisciplinary scholarly attention, with—in our opinion—currently as much a need for empirical research as for conceptual explorations that can guide and stimulate novel modes of thinking. The contributions to this special issue represent a wide range of perspectives, interests, disciplinary backgrounds, and geographical and environmental foci.

In the first part of this special issue, ‘Agenda setting and approaches’ (three papers), Joppa (2015) develops an industry perspective, arguing the need for the building of a community of practice to define key technology challenges and work with a wide variety of partners, in order to harvest the full potential that digital revolution can bring for nature conservation. Maffey et al. (2015) consider what nature conservation can learn from the introduction of digital technology in human development. They derive a charter for conservationists that promotes sensible, collaborative innovation. Galán-Díaz et al. (2015) evaluate digital innovation through partnership between nature conservation organisations and academia. Based on in-depth interviews, they show that besides efficiency benefits, collaboration with academia can bring change in perspectives on technologies with benefits to the partner organisations and staff members therein.

The second part of the issue covers innovation in ‘Monitoring and management’ (three papers). Chapron (2015) proposes a new approach, termed ‘wildlife in the cloud,’ to enable active learning by practitioners from cloud-based ecological models. He argues that this approach has the potential to overcome limitations of desktop-based software (e.g., compatibility, running speed, updates) and illustrates this by presenting an online decision-support tool for moose management in areas with predators in Sweden. Robinson Willmott et al. (2015) present a comprehensive new system (ATOM) that combines thermal imaging with acoustic and ultrasound sensors to continuously monitor bird and bat abundance, flight height, direction, and speed. They illustrate, on the basis of a 16-month-long deployment in the eastern USA, how such technology can be used to generate data which informs the automatic shutting down of (on- and offshore) wind turbines to minimize collision risk. Saito et al. (2015) provide an inspiring example of digital conservation in action in an Asian context. Focussing on various natural areas in Japan, they have developed a system for streaming real-time bird sound from a range of inaccessible locations. Not only does the system allow the (urban) public to connect with nature, the bird sound data also enable ecological surveys to be conducted remotely.

The third part focuses on developments on ‘Citizen science and engagement’ (three papers). Van der Wal et al. (2015a) compare the ability of traditional biological recording schemes and lay citizen science approaches to gather species distribution data. They reveal (for UK bumblebees) that traditional recording generates patchy data which capture the locations of record centres, not a species’ distribution, and call for a further meshing of naturalist and lay initiatives to obtain a national recording capacity. Based on data from eBird, the World’s largest citizen science initiative, Kelling et al. (2015) show how a big data approach to data quality can overcome some of the key problems of analysing inherently noisy records provided by volunteers. Their avant-garde approaches, materialised through close collaboration between computing scientists and ecologists, allow for the creation of species distribution models that accurately estimate patterns of occurrence and abundance for species throughout the year and across the whole of the United States. A similar meshing of disciplines is behind the automated analysis, interpretation and communication of satellite-tag data presented by Van der Wal et al. (2015b). They show that with the help of a relatively simple algorithm and a dedicated website, conservationists and members of the public can be informed immediately of the behaviour of a reintroduced species.

The fourth part is dedicated to ‘Critical appraisal’ of digital technologies (three papers). Newey et al. (2015) provide a practitioners’ account of the many challenges of camera trap use to monitoring wildlife. They present survey results which show that many conservation practitioners use cheaper ‘recreational’ units for research rather than more expensive ‘professional’ equipment, and follow this up with two case studies to provide prospective users with sufficient understanding of the limitations camera-trap technology may pose. Sandbrook (2015) offers a perspective on another increasingly popular monitoring tool, drones. He argues that their potential social impacts can be seriously detrimental for nature conservation, and that there is a need for both empirical research into social impacts as well as self-regulation within the conservation community to guide ethically responsible drone use. Verma et al. (2015) empirically deconstruct the use of new visual media in outreach campaigns of wildlife organisations, and show how these are used to simultaneously enact a ‘microscope’ and the ‘spectacular’ to appeal to both ‘minds’ and ‘hearts’ of the general public as the users of these digital technologies.

In the final contribution, Arts and Van der Wal (2015) present a synthesis of digital innovation in nature conservation. Their analysis of websites and scientific and grey literatures reveals five key areas of application and the perils across those posed by hypes, techno-fix thinking and unverified assumptions related to promise and short-term benefits. They conclude that a re-conceptualisation is desirable of technology as a dual-faced force that can be guided but not always controlled, and call for attention to who benefits from digital conservation and who does not.

With this special issue, “Digital conservation: Understanding the impacts of digital technology on nature conservation,” we hope to accelerate concerted efforts and collective thinking by practitioners, scholars, engineers, activists, programmers, citizen scientists, policy makers and other stakeholders in order to better understand, as well as to steer, the changing nature of conservation in the Digital Age.

## Notes

1. 1. Aichi Biodiversity Targets; 2. Convention on Biological Diversity; 3. Society for Conservation Biology; 4. International Union for Conservation of Nature; 5. United Nations Environment Programme—International Ecosystem Management Partnership; and 6. United Nations Reducing Emissions from Deforestation and Forest Degradation (REDD) and REDD + (which includes the role of conservation, sustainable management of forests and enhancement of forest carbon stocks).
2. 1. Birdlife International; 2. Conservation International; 3. Fauna and Flora International; 4. Nature Conservancy; 5. World Land Trust; and 6. World Wide Fund for Nature.
3. *Protect and restore biodiversity and natural areas*
  Convention on Biological Diversity 1992—‘objectives’ (https://www.cbd.int/convention/articles/default.shtml?a=cbd-01); IUCN 2015—1st key area of work (https://www.iucn.org/what/); Society for Conservation Biology 2015—‘mission (http://www.conbio.org/about-scb/who-we-are); Aichi Biodiversity Targets 2010—strategic goals B and C (http://www.cbd.int/sp/targets/); Fauna and Flora International 2015—‘our mission’ (http://www.fauna-flora.org/about/); World Land Trust 2015—‘our mission’ (http://www.worldlandtrust.org/about/how-we-work); World Wide Fund for Nature 2015—‘mission and vision’ (http://www.worldwildlife.org/about); Conservation International 2015—‘mission’ (http://www.conservation.org/about/Pages/default.aspx#mission); Nature conservancy 2015—‘about us’ (http://www.nature.org/about-us/vision-mission/index.htm); Birdlife International 2015—‘mission’ (http://www.birdlife.org/worldwide/partnership/our-vision-mission-and-commitment).
  *Theorise, collect and analyse data, and model and disseminate scientific findings to support systematic (evidence-based) conservation*
  Society for Conservation Biology 2015; Fauna and Flora International 2015—‘our mission’; World Wide Fund for Nature 2015—‘mission and vision’; Conservation International 2015—‘mission.’
  *Support (local) stakeholder-based conservation and achieve fair and democratic governance and sharing of benefits*
  Convention on Biological Diversity 1992—article 1, IUCN 2015—1st key area of work, Aichi Biodiversity Targets 2010; strategic goal D and E, Fauna and Flora International 2015—‘our mission’ World Land Trust 2015—‘our mission’; World Wide Fund for Nature—‘mission and vision’; Conservation International 2015—‘mission’; Nature conservancy 2015—‘about us’ (http://www.nature.org/about-us/vision-mission/index.htm); Birdlife International 2015—‘mission.’
  *Promote sustainable use and management of natural resources*
  Convention on Biological Diversity 1992—article 1; World Wide Fund for Nature—‘mission and vision’; Conservation International 2015—‘Mission’; Nature conservancy 2015—‘about us’; Birdlife International 2015—‘mission.’ This is often done in the context of global challenges such as climate change, food security and social and economic development (IUCN 2015—3rd key area of work, UN-REDD + Programme—‘about’ (http://www.un-redd.org/AboutREDD/tabid/102614/Default.aspx), Conservation International 2015—‘mission’) and by endorsing conservation aims in public, market and non-profit sectors and/or (inter)national policy and law) cf. Aichi Biodiversity Targets 2010—strategic goal A, UNEP-IEMP 2015—‘mission’ (http://www.unep-iemp.org/vision_mission); World Land Trust 2015—‘our mission’; Conservation International 2015—‘mission’; Nature Conservancy 2015—‘about us.’
4. Strictly speaking, technology is digital where it handles information in binary code (i.e., combinations of the digits 0 and 1, or bits), and it is this very feature which allows for the generation, preservation, processing and transport of vast amounts of information. Where progress in analogue information transition was all about producing higher quality and resolution, digital innovation has largely been about increasing speed and scale of reach. The distinction between the two technologies, however, may be more gradual than absolute, with a digital technology potentially including components that may have analogue modes of information transmission.
5. https://digitalconservation.wordpress.com/.
6. The University of Aberdeen’s Digital Economy Research Hub, funded by Research Councils UK.
7. https://www.abdn.ac.uk/events/digitalconservation/ and http://www.digitalconservation.org/.

## References

[CR1] Arts, K., R. van der Wal, and W.M. Adams. 2015. Digital technology and the conservation of nature. *Ambio* 44(Suppl. 4). doi:10.1007/s13280-015-0705-1. 10.1007/s13280-015-0705-1 PMC4623869 26508352

[CR2] Chapron, G. 2015. Wildlife in the cloud: A new approach for engaging stakeholders in wildlife management. *Ambio* 44(Suppl. 4). doi:10.1007/s13280-015-0706-0. 10.1007/s13280-015-0706-0 PMC4623861 26508343

[CR3] Galán-Díaz, C., P. Edwards, J.D. Nelson, and R. van der Wal. 2015. Digital innovation through partnership between nature conservation organizations and academia: A qualitative impact assessment. *Ambio* 44(Suppl. 4). doi:10.1007/s13280-015-0704-2. 10.1007/s13280-015-0704-2 PMC4623857 26508342

[CR4] Haila Y (2012). Genealogy of nature conservation: a political perspective. Nature Conservation.

[CR6] Joppa, L.N. 2015. Technology for nature conservation: An industry perspective. *Ambio* 44(Suppl. 4). doi:10.1007/s13280-015-0702-4. 10.1007/s13280-015-0702-4 PMC4623870 26508340

[CR7] Kelling, S., D. Fink. F.A. La Sorte, A. Johnston, N.E. Bruns, and W.M. Hochachka. 2015. Taking a ‘Big Data’ approach to data quality in a citizen science project. *Ambio* 44(Suppl. 4). doi:10.1007/s13280-015-0710-4. 10.1007/s13280-015-0710-4 PMC4623867 26508347

[CR8] Mace GM (2014). Whose conservation?. Science.

[CR9] Maffey, G., H. Homans, K. Banks, and K. Arts. 2015. Digital technology and human development: A charter for nature conservation. *Ambio* 44(Suppl. 4). doi:10.1007/s13280-015-0703-3. 10.1007/s13280-015-0703-3 PMC4623862 26508341

[CR10] Newey, S., P. Davidson, S. Nazir, G. Fairhurst, F. Verdicchio, R.J. Irvine, and R. van der Wal. 2015. Limitations of recreational camera traps for wildlife management and conservation research: A practitioner’s perspective. *Ambio* 44(Suppl. 4). doi:10.1007/s13280-015-0713-1. 10.1007/s13280-015-0713-1 PMC4623860 26508349

[CR11] Orton-Johnson K, Prior N (2013). Digital sociology—critical perspectives.

[CR12] Robinson Willmott, J., G.M. Forcey, and L.A. Hooton. 2015. Developing an automated risk management tool to minimize bird and bat mortality at wind facilities. *Ambio* 44(Suppl. 4). doi:10.1007/s13280-015-0707-z. 10.1007/s13280-015-0707-z PMC4623866 26508344

[CR13] Saito, K., K. Nakamura, M. Ueta, R. Kurosawa, A. Fujiwara, H.H. Kobayashi, M. Nakayama, A. Toko, et al. 2015. Utilizing the Cyberforest live sound system with social media to remotely conduct woodland bird censuses in Central Japan. *Ambio* 44(Suppl. 4). doi:10.1007/s13280-015-0708-y. 10.1007/s13280-015-0708-y PMC4623865 26508345

[CR14] Sandbrook, C. 2015. The social implications of using drones for biodiversity conservation. *Ambio* 44(Suppl. 4). doi:10.1007/s13280-015-0714-0. 10.1007/s13280-015-0714-0 PMC4623858 26508350

[CR15] Van der Wal, R., H. Anderson, A. Robinson, N. Sharma, C. Mellish, S. Roberts, B. Darvill, and A. Siddharthan. 2015a. Mapping species distributions: A comparison of skilled naturalist and lay citizen science recording. *Ambio* 44(Suppl. 4). doi:10.1007/s13280-015-0709-x. 10.1007/s13280-015-0709-x PMC4623864 26508346

[CR16] Van der Wal, R., C. Zeng, D. Heptinstall, K. Ponnamperuma, C. Mellish, S. Ben, and A. Siddharthan. 2015b. Automated data analysis to rapidly derive and communicate ecological insights from satellite-tag data: A case study of reintroduced red kites. *Ambio* 44(Suppl. 4). doi:10.1007/s13280-015-0711-3. 10.1007/s13280-015-0711-3 PMC4623868 26508348

[CR17] Verma, A., R. van der Wal, and A. Fischer. 2015. Microscope and spectacle: On the complexities of using new visual technologies to communicate about wildlife conservation. *Ambio* 44(Suppl. 4). doi:10.1007/s13280-015-0715-z. 10.1007/s13280-015-0715-z PMC4623859 26508351

